# Phospho-PRAS40^Thr246^ predicts trastuzumab response in patients with HER2-positive metastatic breast cancer

**DOI:** 10.3892/ol.2014.2744

**Published:** 2014-11-28

**Authors:** KAI YUAN, HONGYAN WU, YULONG WANG, HONGQIANG CHEN, MINGWEN JIAO, RONGZHAN FU

**Affiliations:** Department of Breast Surgery, Shandong Provincial Qianfoshan Hospital, Shandong University, Jinan, Shandong 250014, P.R. China

**Keywords:** proline-rich Akt substrate of 40 kDa, phosphorylation, phosphoinositol-3 kinase, trastuzumab resistance, progression

## Abstract

Resistance to trastuzumab is frequently observed during the treatment of patients with human epidermal growth factor 2 (HER2)-positive metastatic breast cancers. The aim of the present study was to determine if the phosphorylated proline-rich Akt substrate of 40 kDa (phospho-PRAS40^Thr246^), a novel biomarker for phosphoinositol-3 kinase (PI3K) pathway activation, could predict the response of HER2-positive metastatic breast cancers to treatment with trastuzumab. Formalin-fixed, paraffin-embedded tumor tissue samples were retrospectively collected from 55 trastuzumab-treated patients. Next, the expression of phospho-PRAS40^Thr246^ and phosphatase and tensin homolog (PTEN) was assessed by immunohistochemistry. In total, five common phosphoinositol-3 kinase α catalytic subunit mutations, namely E542K, E545K, E545D, H1047R and H1047L, were identified by the amplification-refractory mutation system, using the allele-specific polymerase chain reaction. The activation of the PI3K pathway, as determined by low PTEN expression or the presence of oncogenic PIK3CA mutations, was observed in 49.1% (27 cases) of the 55 HER2-positive metastatic breast cancer tissues. In total, 40% of the tumors were defined as being phospho-PRAS40^Thr246^-positive. Furthermore, the results revealed that phospho-PRAS40^Thr246^ expression was associated with the PI3K pathway activation status and an increased risk of tumor progression in HER2-positive metastatic breast cancer patients who had received trastuzumab-based therapy. Therefore, phospho-PRAS40^Thr246^ expression levels may reflect the PI3K pathway activation status and act as a biomarker for HER2-amplified breast cancer patients who are unlikely to respond to trastuzumab-based therapy.

## Introduction

Human epidermal growth factor receptor 2 (HER2) is amplified in 20–25% of breast cancer cases and is associated with a shorter time to relapse and a reduced overall survival time (1,2). In combination with chemotherapy, the humanized HER2-targeting monoclonal antibody, trastuzumab, demonstrates a marked therapeutic efficacy for treating patients with HER2-expressing metastatic breast cancer. Despite this, a considerable proportion of metastatic HER2-amplified breast cancers fail to respond to, or demonstrate a limited beneficial response to, trastuzumab (3,4). Therefore, an obligation exists to identify novel biomarkers that may predict the response of metastatic breast cancers to trastuzumab.

Previous studies have demonstrated that phosphoinositol-3 kinase (PI3K) activation may be associated with trastuzumab resistance in breast cancer (5). The activation of the PI3K pathway is usually determined by phosphatase and tensin homolog (PTEN) loss and/or activating mutations of phosphoinositol-3 kinase α catalytic subunit (PIK3CA). A number of preclinical studies have demonstrated that PTEN loss and PIK3CA mutation are significantly associated with a poorer trastuzumab-based treatment efficacy in HER2-positive metastatic breast cancers (6). Therefore, the activation status of the PI3K pathway may be used in clinical practice to predict HER2-targeted therapy resistance.

Andersen *et al* (7) revealed that the proline-rich Akt substrate of 40 kDa (PRAS40) phosphorylated at threonine 246 (phospho-PRAS40^Thr246^) was positively correlated with PI3K pathway activation. This identified phospho-PRAS40^Thr246^ as a potential marker of PI3K pathway activation. In addition, the phospho-PRAS40^Thr246^ epitope is highly stable in tissue samples and is therefore suitable for immunohistochemistry (IHC) techniques (7). The present study aimed to investigate whether phospho-PRAS40^Thr246^, as a novel immunostaining marker for PI3K pathway activation, has the potential to predict trastuzumab efficacy in patients with HER2-positive metastatic breast cancer.

## Materials and methods

### Patient materials

Subsequent to obtaining written informed consent, formalin-fixed, paraffin-embedded (FFPE) HER2-overexpressing primary breast carcinoma specimens were retrospectively collected from 55 patients who had developed metastatic breast cancer and had received trastuzumab treatment alone (6 mg/kg alone once every three weeks for one year; n=34) or in combiantion with paclitaxel (175 mg/m^2^, once every three weeks for three months; n=21) between January 2007 and January 2011 at the Shandong Provincial Qianfoshan Hospital (Jinan, China). This study was approved by the ethics committee of Shandong Provincial Qianfoshan Hospital, Shandong University (Jinan, China). Complete data on tumor characteristics, treatment details and follow-up results of disease progression were obtained for all cases. Prior to the study, no patient had received trastuzumab-based neoadjuvant therapy. The tumors were identified as HER2-positive by the overexpression of HER2, as detected by IHC, and/or HER2 gene amplification using fluorescence *in situ* hybridization. According to standard clinical instructions, the expression of the estrogen and progesterone receptors was evaluated by IHC, and expression data were retrospectively reviewed and extracted from the medical records. An increase in the diameters of the existing lesions by ≥20%, or the appearance of any new lesion, was defined as progressive disease (8). The time-to-progression (TTP) was calculated starting from the initiation of trastuzumab-based treatment until the time of disease progression. The clinical and pathological characteristics are shown in Table I.

### IHC

IHC was performed using 4-μm thick FFPE breast cancer sections. Subsequent to a 10-min incubation in microwave-based antigen-retrieval solutions (0.1 M sodium citrate), immunostaining was performed using a primary monoclonal rabbit anti-human PTEN antibody (dilution 1:50; Cell Signaling Technology, Beverly, MA, USA) and a polyclonal rabbit anti-human phospho-PRAS40^Thr246^ antibody (dilution 1:100; Cell Signaling Technology). The negative control group received rabbit immunoglobulin G (1:50 for PTEN; 1:100 for phospho-PRAS40^Thr246^; Cell Signaling Technology) at the same concentration. The immunohistochemical results of PTEN expression were evaluated semi-quantitatively using the immunoreactive score (IRS). The IRS was achieved by multiplying the staining intensity (0, negative; <1, weak; 2, moderate; and 3, strong) by the percentage of positive cells (0, 1%; 1, 1–10%; 2, 11–50%; 3, 51–80%; and 4, >80%) within each whole tissue section. An IRS score of ≤3 was defined as PTEN loss (9). The phospho-PRAS40^Thr246^ immunostaining intensity of each tumor sample was interpreted by an H score system, which was derived by multiplying the fraction of positively-stained tissue (%) by the intensity of the staining (1, weak; 2, moderate; and 3, strong). An H score of ≥100 defined cases as phospho-PRAS40^Thr246^-positive (7).

### Detection of PIK3CA mutations

The genomic DNA of FFPE sections consisting of ≥50% tumor cells was extracted using an E.Z.N.A FFPE DNA Isolation kit (Omega Bio-Tek, Norcross, GA, USA), according to the manufacturer’s instructions. In total, five common PIK3CA mutations, namely E542K, E545K in exon 9, E545D, H1047R and H1047L in exon 20, were detected by the amplification-refractory mutation system, allele-specific polymerase chain reaction, using an AmoyDx™ PIK3CA Five Mutations Detection kit (Amoy Diagnostics Co., Ltd., Haicang, China), as previously described (10).

### Statistics

The association between phospho-PRAS40^Thr246^ expression and other clinical or pathological characteristics was determined using the χ^2^ test. The Kaplan-Meier method was used to plot the TTP data. The differences between the groups were analyzed using the log-rank test. The univariate and multivariate analyses of predictive factors were performed using Cox’s proportional hazard regression. All tests were performed using MedCalc 11.4 software (MedCalc, Ostend, Belgium). P<0.05 was considered to indicate a statistically significant difference.

## Results

**Figure 1:**
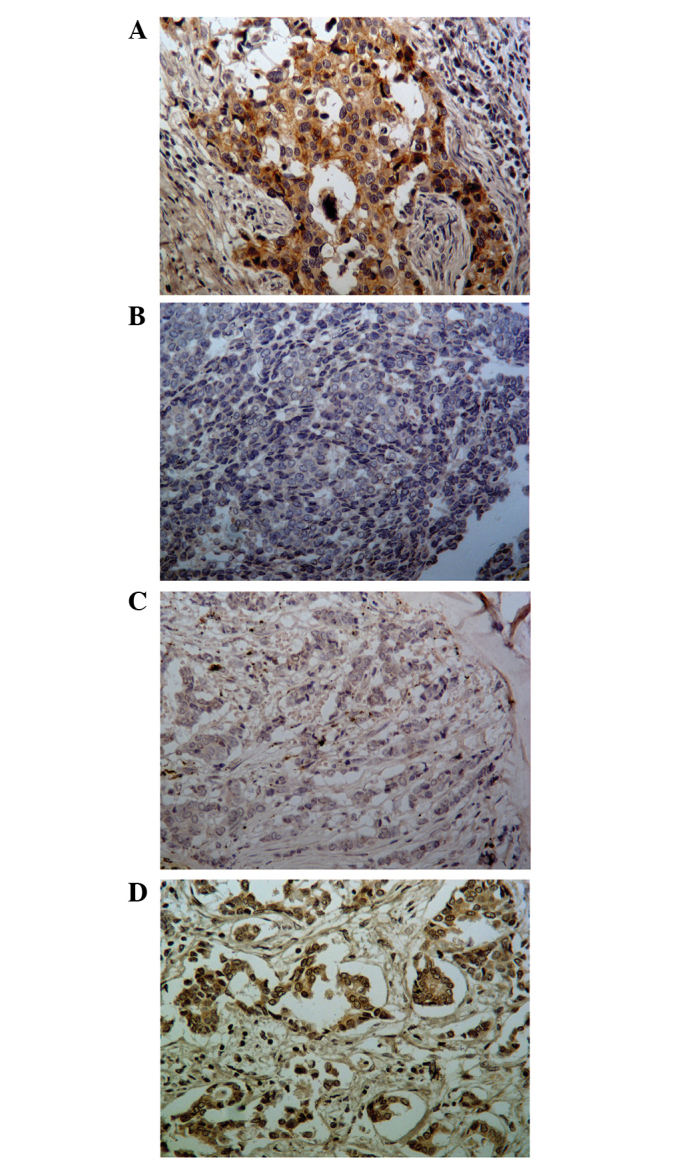
Immunostaining images revealing phosphorylated proline-rich Akt substrate of 40 kDa (phospho-PRAS40^Thr246^) and phosphatase and tensin homolog (PTEN) expression in human epidermal growth factor 2-positive metastatic breast cancers. (A) Phospho-PRAS40^Thr246^-positive staining, (B) phospho-PRAS40^Thr246^-negative staining, (C) PTEN-negative staining and (D) PTEN-positive staining (magnification, ×200).

In the present study, IHC was used to detect phospho-PRAS40^Thr246^ expression in HER2-positive metastatic breast cancer tissues. The specificity and suitability of the phospho-PRAS40^Thr246^ antibody used for the IHC has been validated by previous studies (11,12). As revealed in Fig. 1, phospho-PRAS40^Thr246^ was expressed diffusely throughout the cytoplasm of the breast cancer cells at varying intensities, and demonstrated an occasional nuclear location. Based on previous studies, an H score of ≥100 was used as the criterion for the positive expression of phospho-PRAS40^Thr246^. Using this range, a total of 22 cases (40.0%) of HER2-positive breast cancer tumors were defined as phospho-PRAS40^Thr246^-positive. The clinicopathological variables of the patients were not significantly associated with the phospho-PRAS40^Thr246^ expression levels (Table I).

In addition, the PI3K pathway activation status of the same set of HER2-positive breast cancer tumors was investigated. In total, PTEN loss was demonstrated in 34.5% (19/55) of the HER2-positive breast cancer tissues. Furthermore, seven mutations in exon 20 (H1047R) and three in exon 9 (E542K and E545K) were identified, which corresponded to a PIK3CA mutation frequency of 18.2%. In agreement with previous findings (5,13), with the exception of two cases, PTEN loss and PIK3CA mutation status were not present within the same tumor samples in the present study. According to the previously established criteria (5), patients were classified as having an activated (with either PIK3CA mutants or low PTEN expression; n=27) or an unactivated PI3K pathway status (with PIK3CA mutant type and high PTEN expression; n=28). A significant association was identified between phospho-PRAS40^Thr246^ expression and PI3K pathway status, and PTEN loss alone, but not with PIK3CA mutation alone (Table II).

**Figure 2:**
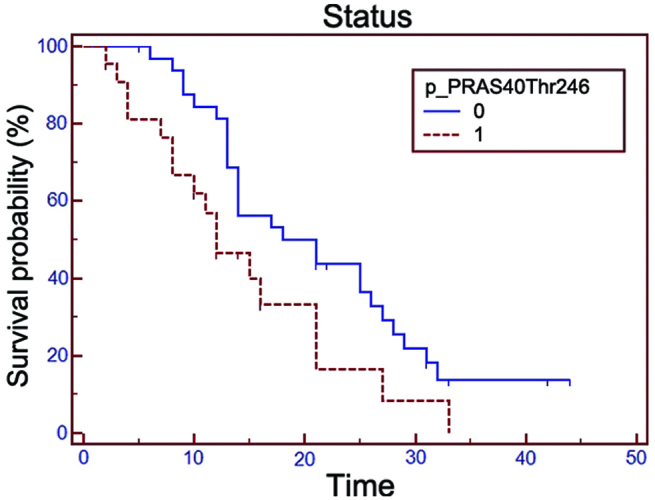
Kaplan-Meier curves of time-to-progression according to phosphorylated proline-rich Akt substrate of 40 kDa (phospho-PRAS40^Thr246^) expression in HER2-positive metastatic breast cancers (1, positive; 0, negative). A significant difference was observed between the phospho-PRAS40^Thr246^-positive and -negative groups (P=0.031). Differences between the two groups were evaluated using the log-rank test.

Univariate analysis was used to determine the association between disease progression and PI3K pathway activation status following trastuzumab-based treatment in HER2-positive metastatic breast cancers, as determined by different marker sets. As revealed in Table II, patients with PIK3CA mutations and/or PTEN loss were at an increased risk for disease progression compared with other subgroups. Furthermore, patients with positive phospho-PRAS40^Thr246^ expression exhibited a significantly shorter TTP following trastuzumab-based treatment than those with negative expression (Fig. 2). The hazard ratios (HR), which were based on multivariate Cox regression analysis, and were adjusted for other significant predictors (grade), revealed that positive phospho-PRAS40^Thr246^ expression was an independent and significant risk factor for disease progression (HR, 2.081; 95% confidence interval, 1.113–3.890; P=0.022; Table II)

## Discussion

Hyperactivation of the PI3K pathway, as determined by PIK3CA mutations or low PTEN expression, was observed in ~50% of the HER2-positive metastatic breast cancers in the present study. In agreement with previous studies (14,15), PI3K hyperactivation contributed to an increased risk of disease progression following trastuzumab treatment.

In addition to PIK3CA mutations and low PTEN expression, a panel of biomarkers [including topoisomerase IIα (16), MET oncogene, hepatocyte growth factor (17), phospho-AKT^Ser473^ and phospho-p70S6^KThr389^ (8)] have been revealed to be associated with PI3K pathway activation, and were proposed as candidate biomarkers to predict trastuzumab resistance in HER2-positive metastatic breast cancers. Despite this, the markers demonstrate limited associations with trastuzumab efficacy in clinical sets.

Phospho-PRAS40^Thr246^ is a novel downstream target of the PI3K/Akt signaling pathway (18). In a recent study, phospho-PRAS40^Thr246^ expression was positively correlated with PI3K pathway activation and predicted PI3K pathway inhibitor sensitivity in triple-negative breast tumor tissues and a PTEN-deficient mouse prostate tumor model (7). Due to a high epitope stability, phospho-PRAS40^Thr246^ has an improved clinical translation potential compared with other PI3K pathway downstream targets, such as AKT^Thr308^ (7). A previous study identified that phospho-PRAS40^Thr246^ was overexpressed in ~40% of primary breast cancer samples, and as an independent prognostic factor, was associated with slower disease progression (12). The present study indicated that phospho-PRAS40^Thr246^ expression was significantly associated with PIK3CA mutations and low PTEN expression. In addition, more than twice as many patients were identified at an increased risk for disease progression. Therefore, the analysis of phospho-PRAS40^Thr246^ expression levels could reflect PI3K pathway activation status and present a novel biomarker to identify HER2-amplified breast cancers that are unlikely to respond to trastuzumab-based therapy.

A previous study demonstrated that the PI3K inhibitor, GDC-0941, decreased the phosphorylation of PRAS40 and was highly efficacious in the treatment of trastuzumab-resistant cells and tumors (19). These results, together with the association between phospho-PRAS40^Thr246^ expression and trastuzumab resistance identified in the present study, suggest that phospho-PRAS40^Thr246^ may be a candidate for predicting trastuzumab responses. However, the potential mechanism has not previously been investigated.

The present study revealed the significance of phospho-PRAS40^Thr246^ in identifying HER2-positive breast cancer patients with a high risk of disease progression following trastuzumab-based therapy. In addition, phospho-PRAS40^Thr246^ may possess an important role in predicting the development of trastuzumab resistance. The exact molecular mechanism underlying the effect of phospho-PRAS40^Thr246^ expression levels on trastuzumab resistance requires further investigation in order to be clinically beneficial.

## Figures and Tables

**Table I:** Association between phospho-PRAS40^Thr246^ expression, PI3K activation markers and clinicopatholgical characteristics.

	Phospho-PRAS40^Thr246^		
		
Characteristics	Negative, n	Positive, n	P-value
Age, years			0.319
Premenopausal	13	5	
Postmenopausal	20	17	
Grade			0.869
1–2	17	10	
3	16	12	
ER status			0.407
Negative	20	10	
Positive	13	12	
PR status			0.860
Negative	23	14	
Positive	10	8	
PIK3CA mutation			0.284
Negative	29	16	
Positive	4	6	
PTEN loss			0.024
Negative	26	10	
Positive	7	12	
PIK3CA mutants or low PTEN			0.001
No	24	4	
Yes	9	18	

Phospho-PRAS40^Thr246^, phosphorylated proline-rich Akt substrate of 40 kilodaltons; ER, estrogen receptor; PR, progesterone receptor; PTEN, phosphatase and tensin homolog; PIK3CA, phosphoinositol-3 kinase α catalytic subunit.

**Table II:** Univariate and multivariate analysis for overall survival.

	Univariate analysis	Multivariate analysis	
		
Characteristics	P-value	HR (95% CI)	P-value
Age, years	0.805		
Grade	0.016	2.253 (1.187–4.274)	0.013
ER status	0.411		
PR status	0.487		
PIK3CA mutation	0.163		
PTEN loss	0.070		
PI3K activation	0.013		
Phospho-PRAS40^Thr246^	0.031	2.081 (1.113–3.890)	0.022

HR, hazard ratio; CI, confidence interval; ER, estrogen receptor; PR, progesterone receptor; PIK3CA, phosphoinositol-3 kinase α catalytic subunit; PTEN, phosphatase and tensin homolog; PI3K, phosphoinositol-3 kinase; Phospho-PRAS40^Thr246^, phosphorylated proline-rich Akt substrate of 40 kDa.
